# Whole‐genome sequencing elucidates the species‐wide diversity and evolution of fungicide resistance in the early blight pathogen *Alternaria solani*

**DOI:** 10.1111/eva.13350

**Published:** 2022-02-22

**Authors:** Severin Einspanier, Tamara Susanto, Nicole Metz, Pieter J. Wolters, Vivianne G.A.A. Vleeshouwers, Åsa Lankinen, Erland Liljeroth, Sofie Landschoot, Žarko Ivanović, Ralph Hückelhoven, Hans Hausladen, Remco Stam

**Affiliations:** ^1^ 9184 Chair of Phytopathology Technical University of Munich Freising Germany; ^2^ Plant Breeding Wageningen University and Research Wageningen The Netherlands; ^3^ Department of Plant Protection Swedish University of Agricultural Sciences Lomma Sweden; ^4^ Department of Plants and Crops University of Ghent Ghent Belgium; ^5^ 229804 Institute for Plant Protection and Environment Belgrade Serbia; ^6^ 9184 Plant Technology Centre Technical University of Munich Freising Germany

**Keywords:** agriculture, alternaria solani, fungicide resistance, plant pathology, population genetics – empirical, potato

## Abstract

Early blight of potato is caused by the fungal pathogen *Alternaria solani* and is an increasing problem worldwide. The primary strategy to control the disease is applying fungicides such as succinate dehydrogenase inhibitors (SDHI). SDHI‐resistant strains, showing reduced sensitivity to treatments, appeared in Germany in 2013, shortly after the introduction of SDHIs. Two primary mutations in the SDH complex (*SdhB*‐H278Y and *SdhC*‐H134R) have been frequently found throughout Europe. How these resistances arose and spread, and whether they are linked to other genomic features, remains unknown. For this project, we performed whole‐genome sequencing for 48 *A*. *solani* isolates from potato fields across Europe to better characterize the pathogen's genetic diversity in general and understand the development and spread of the genetic mutations that lead to SDHI resistance. The isolates can be grouped into seven genotypes. These genotypes do not show a geographical pattern but appear spread throughout Europe. We found clear evidence for recombination on the genome, and the observed admixtures might indicate a higher adaptive potential of the fungus than previously thought. Yet, we cannot link the observed recombination events to different *Sdh* mutations. The same *Sdh* mutations appear in different, non‐admixed genetic backgrounds; therefore, we conclude they arose independently. Our research gives insights into the genetic diversity of *A*. *solani* on a genome level. The mixed occurrence of different genotypes, apparent admixture in the populations, and evidence for recombination indicate higher genomic complexity than anticipated. The conclusion that SDHI tolerance arose multiple times independently has important implications for future fungicide resistance management strategies. These should not solely focus on preventing the spread of isolates between locations but also on limiting population size and the selective pressure posed by fungicides in a given field to avoid the rise of new mutations in other genetic backgrounds.

## 1 INTRODUCTION

Early blight is one of the major diseases in potato‐growing areas. The causal agent of early blight is the fungal pathogen *Alternaria solani*. If the weather conditions are favourable and *A*. *solani* remains uncontrolled, yield losses can reach up to 40% (Kapsa, 2004; Leiminger & Hausladen, 2012, 2014). Typical symptoms of potato early blight are dark brown to black lesions with concentric rings. In the beginning, the necrotic area is often surrounded by a yellow, chlorotic halo (Rotem, 1994). Due to climatic changes Delgado‐Baquerizo et al. (2020) predicted a global increase of soil‐borne pathogens, for example, *Alternaria* spp., which would imply increasing disease pressure in the field. The potato crop is the fourth most important field crop in European agriculture based on yield (tons/year), after wheat, maize, and barley (Eurostat 2020). To guarantee the yield, farmers adhere to good agricultural practices, for example, crop rotation and sufficient nutrient supply, but in most cases the application of fungicides is needed.

For early blight control, three main fungicide groups are available: the Quinone outside Inhibitors (QoIs), the Succinate Dehydrogenase Inhibitors (SDHIs), and the Demethylation Inhibitors (DMIs), of which QoIs and SDHIs are most widely used and until recently considered most effective.

The development of resistance or reduced sensitivity to the compounds has been reported in *A*. *solani* and other fungi for all of these fungicide groups. DMI resistance appears to be related to changes in expression of the target site, Cyp51, possibly linked with a mutation (Zhang et al., 2020). Mutations in *Cyp51* genes are common in other pathogens to which DMIs are applied (Blake et al., 2018; Pereira et al., 2017). For the other two fungicide groups, mutations in the pathogen target sites have already been confirmed in *A*. *solani*. The F129L mutation in the cytochrome b of the mitochondrial electron transport complex III (Bartlett et al., 2002) frequently occurs in the field (Leiminger and Hausladen, 2014; Odilbekov et al., 2016). Leiminger and Hausladen (2014) showed a reduced efficacy of azoxystrobin (a QoI) by almost 50% for the F129L‐mutant isolates *in vivo* in comparison to wild‐type isolates. Concerning the SDHIs, several different mutations have been identified in the subunits b, c, and d of the *sdh*‐gene in the mitochondrial electron transport complex II: *Sdh*B‐H278Y, *Sdh*B‐H278R, *Sdh*C‐H134R, *Sdh*C‐H134Q, *Sdh*D‐D123E, *Sdh*D‐H133R (Mallik et al., 2014; Metz et al., 2019). For these SDH mutations, a negative impact on fungicide efficiency has also been shown in several studies (Landschoot et al., 2017; Metz et al., 2019).

In a European study, 70% of *A*. *solani* isolates sampled between 2014 and 2015 contained one of the SDHI mutations and 40% also had the cytochrome b F129L mutation, leading to QoI resistance and thus possessing a dual fungicide resistance (Landschoot et al., 2017). A more recent, long‐term Swedish field study showed that nearly all Swedish isolates now carry the F129L mutation (Edin et al., 2019). In Germany also nearly all samples carry the F129L mutation and 43% show mutations in SDH subunits (Nottensteiner et al., 2019). In the United States, a study of over 1000 isolates revealed that between 2013 and 2015 the presence of the F129L mutation rose from 92 to 99% and mutations in any of the SDH subunits were also present in 99% of the isolates (Bauske et al., 2017).

The genetic diversity of *A*. *solani* in the field is relatively understudied, possibly because *A*. *solani* historically has been described as an asexual species and limited variation was expected. Yet, considerable genetic diversity was found in South African isolates using random amplified microsatellite (RAMS) primers (van der Waals et al., 2004). Random amplification of polymorphic DNA (RAPD) profiling confirmed this surprisingly high genetic diversity of field isolates in Germany (Leiminger et al., 2016) or Wisconsin (Weber & Halterman, 2012). Also on tomato in India, a marker‐based study revealed higher within and between state diversity in *A*. *solani* than anticipated by the authors (Upadhyay et al., 2019). Barcode sequencing revealed the presence of multiple *A*. *solani* haplotypes in a single field (Adhikari et al., 2020).

Odilbekov found that the genetic composition of the pathogen populations in the field changed after fungicide treatment. Their study revealed an increase of genotypes with reduced sensitivity over time (Odilbekov et al., 2019). However, the use of Amplified Fragment Length Polymorphisms (AFLP) limited the resolution of the genetic diversity analysis, and only two main genotypes could be described. Genome‐wide sequencing approaches, such as Genotyping By Sequencing (GBS) or full genome sequence analyses, are very powerful tools that are able to reveal genomic variation that previously remained hidden (Everhart et al., 2020). Indeed, a GBS study revealed that microevolutionary factors might play an important role in population structure of various *Alternaria* spp. (Adhikari et al., 2019).

Whereas fungicide resistance is on the rise in *A*. *solani*, it is not clear whether the causal mutations occur in one or few genetic backgrounds and spread or whether they arose multiple times independently in different genetic backgrounds. Understanding such microevolutionary forces at play in and between populations and their roles in fungicide resistance evolution is important. If mutations arise only once and then spread, this should have implications for management practices, for instance on transportation and movement of tubers and infected material.

Here we combine a genomic diversity study with analyses of fungicide resistance targets. We make use of the recently published *A*. *solani* reference genome (Wolters et al., 2018) and a Europe‐wide selection of *A*. *solani* samples to study the genetic diversity of the isolates in general and the occurrence of SDHI resistance in a genomic context.

## 2 METHODS

### 2.1 Fungal material

**FIGURE 1:**
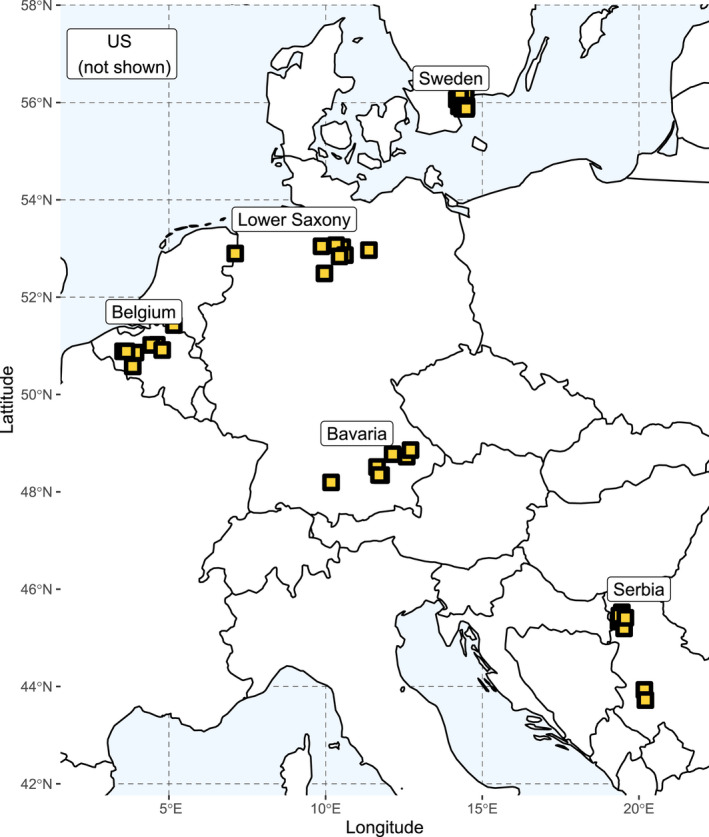
Overview of the sampling locations used in this study

Forty‐eight different isolates were collected from five different localities across Europe (Figure 1). All samples were reported to possess the F129L mutation in cytochrome b, associated with QoI resistance. The status of SDHI resistance were partly mixed (reported wild‐type isolates as well as reported mutants) and partly unknown. All isolates were collected between 2014 and 2017 when SDHI resistance was reported to be on the rise, but not fully spread. This would allow screening of genetic backgrounds prior eventual fungicide‐related bottlenecks. Isolates DE_NM014 – DE_NM020 were collected from potato fields in Bavaria, Germany, DE_NM006 – DE_NM013 from fields in Lower Saxony in Germany. SE_EL001 – SE_EL012 isolates originate from Southern Sweden, BE_SL001 – BE_SL008 from fields in Belgium, and RS_ZI001 – RS_ZI008 were collected from two localities in Serbia. US_JW001 – US_JW004 and US_NM022 were collected in the United States. A detailed overview can be found in Table 1. All isolates were sampled in their respective fields from symptomatic potato leaves. Leaves were dried between tissue paper and surface sterilized before placement on Synthetic Nutrient Agar (SNA) (0.2 g/L glucose, 0.2 g/L sucrose, 0.5 g/L MgSO_4_‐7H_2_O, 0.5 g/L KCl, 1.0 g/L KH_2_PO_4_, 1.0 g/L KNO_3_, 22.0 g/L agar; 600 µl/L 1 M NaOH). A single spore was collected from each isolate and propagated under sterile condition for further usage.

**TABLE 1:** Overview of the isolates used in this study

CODE	Name	SRA Number	Original Name	Locality	Country	Year of Isolation	SDHI Reported	SDHI Detected	QoI	Haplotype Adhikari	Haplotype Multigene	GT group	Potato	Latitude	Longitude	Sequence depth
BE	SL001	SRS9529518	15.19s	Vollezele	Belgium	2015	Unknown	H134R	F129L	AsAlHs4	MG_H1	GT7	Bintje	50.760587	4.025269	54
BE	SL002	SRS9529519	14.23s	Leefdaal	Belgium	2014	Unknown	wt	F129L	unknown	MG_H1	no GT	Bintje	50.848069	4.604352	87
BE	SL003	SRS9529520	15.43s	Wannegem‐Lede	Belgium	2015	Unknown	H134R	F129L	AsAlHs4	MG_H2	GT7	Bintje	50.891398	3.55009	49
BE	SL004	SRS9529521	15.183s	Leefdaal	Belgium	2015	Unknown	wt	F129L	AsAlHs4	MG_H1	GT2	Bintje	50.848069	4.604352	79
BE	SL005	SRS9529522	15.186s	Kasterlee	Belgium	2015	Unknown	H278Y	F129L	AsAlHs4	MG_H1	GT4	Fontane	51.252769	4.959916	80
BE	SL006	SRS9529523	15.235s	Merksplas	Belgium	2015	Unknown	H278R	F129L	AsAlHs4	MG_H2	GT6	Bintje	51.371243	4.877228	42
BE	SL007	SRS9529524	15.1s	Leefdaal	Belgium	2015	wt	H278Y	F129L	AsAlHs4	MG_H2	GT6	Bintje	50.848069	4.604352	69
BE	SL008	SRS9529525	15.20s	Herne	Belgium	2015	wt	wt	F129L	AsAlHs4	MG_H2	GT2	Bintje	50.724535	4.040529	44
DE	NM006	SRS9529526	676_1	Rupennest bei Lathen	Lower Saxony	2015	wt	wt	F129L	AsAlHs4	MG_H2	GT2	Unknown	52.87937461	7.308617612	54
DE	NM007	SRS9529527	687_1	Immensen/Lehrte	Lower Saxony	2015	H134R	H134R	F129L	AsAlHs4	MG_H1	GT7	Unknown	52.38191091	10.01725194	55
DE	NM008	SRS9529529	691_1	Hamerstorf	Lower Saxony	2015	H134R	H134R	F129L	AsAlHs4	MG_H1	GT7	Unknown	52.87658323	10.42499062	52
DE	NM009	SRS9529530	692_1	Gusborn	Lower Saxony	2015	H134R	H134R	F129L	AsAlHs4	MG_H1	GT7	Unknown	53.07766577	11.19777163	43
DE	NM010	SRS9529531	711_2	Hamerstorf	Lower Saxony	2015	wt	H278Y	F129L	AsAlHs4	MG_H1	GT3	Unknown	52.87658323	10.42499062	55
DE	NM011	SRS9529532	720_3	Hamerstorf	Lower Saxony	2015	H278Y	H278Y	F129L	AsAlHs4	MG_H1	GT3	Unknown	52.87658323	10.42499062	52
DE	NM012	SRS9529533	754_2	Solltau	Lower Saxony	2015	H278Y	H278Y	F129L	AsAlHs4	MG_H1	GT3	Unknown	52.99580915	9.856700578	52
DE	NM013	SRS9529534	774_1	Hamerstorf	Lower Saxony	2015	H278Y	H278Y	F129L	AsAlHs4	MG_H1	GT3	Unknown	52.87658323	10.42499062	54
DE	NM014	SRS9529535	732_1	Laberweinting	Bavaria	2015	H134R	H134R	F129L	AsAlHs4	MG_H1	GT7	Unknown	48.78143934	12.31529157	56
DE	NM015	SRS9529536	736_1	Hallbergmoos	Bavaria	2015	wt	wt	F129L	AsAlHs4	MG_H2	GT6	Unknown	48.31047191	11.72974033	54
DE	NM016	SRS9529537	737_1	Ismaning	Bavaria	2015	H278Y	H278Y	F129L	AsAlHs4	MG_H1	GT4	Unknown	48.24057911	11.70475989	52
DE	NM017	SRS9529538	739_3	Feldkirchen	Bavaria	2015	wt	wt	F129L	AsAlHs4	MG_H2	GT6	Unknown	48.83017658	12.5302467	54
DE	NM018	SRS9529540	746_1	Aiterhofen	Bavaria	2015	wt	wt	F129L	AsAlHs4	MG_H1	GT3	Unknown	48.8417768	12.62818583	56
DE	NM019	SRS9529496	749_3	Freising	Bavaria	2015	wt	N75S	F129L	AsAlHs4	MG_H2	GT6	Unknown	48.39358972	11.70637674	46
DE	NM020	SRS9529495	615	Kirchheim	Bavaria	2014	wt	wt	F129L	AsAlHs4	MG_H1	GT3	Unknown	48.32771814	10.30486316	56
RS	ZI001	SRS9529505	45–1	Bački Maglić	Serbia	2016	wt	wt	F129L	AsAlHs4	MG_H1	GT1	Brooke	45.367993	19.513177	67
RS	ZI002	SRS9529506	45–12	Bački Maglić	Serbia	2016	wt	wt	F129L	AsAlHs4	MG_H1	GT1	VR 808	45.367993	19.513177	69
RS	ZI003	SRS9529517	54–1	Guča	Serbia	2016	wt	wt	F129L	AsAlHs4	MG_H1	GT6	Marabel	43.76386	20.234928	61
RS	ZI004	SRS9529528	54–10	Guča	Serbia	2016	wt	wt	F129L	AsAlHs4	MG_H1	GT6	Marabel	43.76386	20.234928	64
RS	ZI005	SRS9529539	45–4	Bački Maglić	Serbia	2016	Unknown	wt	F129L	AsAlHs4	MG_H1	GT4	Brooke	45.367993	19.513177	83
RS	ZI006	SRS9529501	45–13	Bački Maglić	Serbia	2016	Unknown	wt	F129L	AsAlHs4	MG_H1	GT4	VR808	45.367993	19.513177	60
RS	ZI007	SRS9529500	47–10	Bački Maglić	Serbia	2016	Unknown	wt	F129L	AsAlHs4	MG_H1	GT4	Lady Claire	45.367993	19.513177	80
RS	ZI008	SRS9529502	38–1	Bački Maglić	Serbia	2016	Unknown	wt	F129L	AsAlHs4	MG_H1	GT4	Opal	45.367993	19.513177	85
SE	EL001	SRS9529503	2017_152.1	Nymö	Sweden	2017	wt	wt	F129L	AsAlHs4	MG_H1	GT1	Kuras	56.017577	14.332252	69
SE	EL002	SRS9529504	2017_152.3	Nymö	Sweden	2017	wt	wt	F129L	AsAlHs4	MG_H1	GT1	Kuras	56.017577	14.332252	84
SE	EL003	SRS9529507	2017_212.2	Nymö	Sweden	2017	wt	wt	F129L	AsAlHs4	MG_H1	GT2	Kuras	56.017577	14.332252	66
SE	EL004	SRS9529509	2017_213.1	Nymö	Sweden	2017	wt	wt	F129L	AsAlHs4	MG_H1	GT2	Kuras	56.017577	14.332252	71
SE	EL005	SRS9529508	2017_131.4	Nymö	Sweden	2017	H134R	H134R	F129L	AsAlHs4	MG_H1	GT7	Kuras	56.017577	14.332252	66
SE	EL006	SRS9529510	2017_134.1	Nymö	Sweden	2017	H134R	H134R	F129L	AsAlHs4	MG_H1	GT1	Kuras	56.017577	14.332252	69
SE	EL007	SRS9529511	2017_144.2	Nymö	Sweden	2017	H134R	H134R	F129L	AsAlHs4	MG_H1	GT7	Kuras	56.017577	14.332252	79
SE	EL008	SRS9529512	2017_152.4	Nymö	Sweden	2017	H134R	H134R	F129L	AsAlHs4	MG_H1	GT7	Kuras	56.017577	14.332252	65
SE	EL009	SRS9529513	2017_124.2	Nymö	Sweden	2017	H278Y	H278Y	F129L	AsAlHs4	MG_H1	GT4	Kuras	56.017577	14.332252	67
SE	EL010	SRS9529514	2017_132.2	Nymö	Sweden	2017	H278Y	H278Y	F129L	AsAlHs4	MG_H1	GT3	Kuras	56.017577	14.332252	79
SE	EL011	SRS9529515	2017_142.2	Nymö	Sweden	2017	H278Y	H134R	F129L	AsAlHs4	MG_H1	GT7	Kuras	56.017577	14.332252	66
SE	EL012	SRS9529516	2017_151.3	Nymö	Sweden	2017	H278Y	H278Y	F129L	AsAlHs4	MG_H1	GT2	Kuras	56.017577	14.332252	76
US	JW001	SRS9529493	CONR4D	Unknown	Idaho	2015	Unknown	H134R	F129L	AsAlHs4	MG_H1	GT5	Unknown	Unknown	Unknown	53
US	JW002	SRS9529494	CONRI4	Unknown	Idaho	2015	Unknown	H134R	F129L	AsAlHs4	MG_H1	GT1	Unknown	Unknown	Unknown	54
US	JW003	SRS9529497	OONR2J	Unknown	Idaho	2015	Unknown	I280V	F129L	AsAlHs4	MG_H1	GT5	Unknown	Unknown	Unknown	50
US	JW004	SRS9529498	T2R3B	Unknown	Idaho	2015	Unknown	H134R	F129L	AsAlHs4	MG_H1	GT5	Unknown	Unknown	Unknown	49
US	NM022	SRS9529499	628	Unknown	unknown	2015	H134R	H134R	F129L	AsAlHs4	MG_H1	GT7	Unknown	Unknown	Unknown	52

Note

Observed and reported mutations refer to mutations in different SDHI subunits. H278Y and I280V occur in subunit B, H134R in Subunit C, and N75S in subunit D. Unknown indicates that a mutation was assumed in these isolates, but the exact nature was not established prior this study.

### 2.2 DNA extraction and sequencing

For high‐quality DNA extraction, 200 ml of potato dextrose broth was inoculated with small agar plugs from 10 to 14 days old *A*. *solani* cultures, grown on SNA. Liquid cultures were incubated for 48 hours at 28°C on a rotary shaker (110 rpm). Afterwards, mycelium was filtered through a cheese cloth and squeezed to remove most of the liquid. After freeze drying, mycelium was grinded with liquid nitrogen and a bit of clean sea sand to break the cells most efficiently. 50–100 mg of lyophilized powder was added to 1 ml of DNA extraction buffer (containing extraction buffer (0.35 M sorbitol, 0.1 M Tris‐HCl pH7.5, 5 mM EDTA), nucleic lysis buffer (2% CTAB, 2 M NaCl, 0.2 M Tris‐HCl pH7.5, 50 mM EDTA), and sarkosyl (10%) in a ratio of 2.5:2.5:1), 20µl proteinase K (25 mg/ml), and 20µl RNAse A (20 mg/ml) in a 2 ml tube. Everything was mixed well and incubated for 1hour at 60°C – inverting the tubes occasionally to gently mix the content. Afterwards samples were centrifuged for 10 minutes at maximum speed, and the supernatant was transferred into a new tube. An equal amount of PCI (Phenol:Chloroform:Isoamylalcohol (24:24:1)) was added to the tube and incubated overnight on a rotary shaker at 4°C (gentle rotation to avoid damaging DNA). On the following day, samples were spun down for 15 minutes at maximum speed and upper phase was transferred to a new tube. An equal amount of SEVAG (Chloroform:Isoamylalcohol (24:1)) was added and tubes were incubated for at least 1 hour on a rotary shaker at 4°C (gentle rotation). In a next step, samples were centrifuged for 10 minutes at maximum speed and supernatant was transferred to a new tube. For precipitation, 0.7 volume of isopropanol (room temperature) was added and samples were spun down for 15 minutes at 9,600 *g*. After washing the DNA pellet with 70% ethanol, remaining liquid was removed by pipetting. To entirely remove the ethanol, tubes were placed in a heating block at 37°C to let them dry completely. DNA pellet was dissolved in 100 µl distilled water and stored at 4°C. A Qubit was used to determine the quantity of the extracted DNA. Sequencing libraries were prepared by BGI Ltd (Hong Kong) using their proprietary protocol. The sequencing was done on the DNBSEQ platform, using paired end 150 bp reads, aiming for up to 3 Gb of data per sample.

### 2.3 Mapping and SNP processing

The quality of the raw sequence data was checked using FastQC version 0.11.5 Andrews, 2016). The Burrows‐Wheeler alignment tool (version 1) (Li & Durbin, 2009) was used to map all samples to our reference genome NL03003 (Wolters et al., 2018). Reads deduplication was performed using the picard tool Markduplicate (version 2.19.2). Subsequently, the single‐nucleotide polymorphisms (SNPs) were called using GATKs Haplotype‐Caller (version 4.1.7.0) in GVCF mode (McKenna et al., 2010) with default settings, except for having ploidy set to one. The SNPs were filtered using GATK with modified parameters compared to the recommended filtering criteria. SNPs were filtered out when they met the following parameters: low mapping quality rank sum test (MQRankSum < −12.5), low quality by depth (QD <2.0), low read pos rank sum test (ReadPosRankSum < −8.0), high Fisher strand difference (FS >60.0), low RMS mapping quality (MQ <40.0), high strand odds ratio (SOR >3.0), high haplotype score (HaplotypeScore >13.0). Additionally, the insertion or deletions (indels) were filtered out using GATK with the following parameters: low quality depth (QD <2.0), low read pos rank sum test (ReadPosRankSum < −20.0), and high Fisher strand difference (FS >200.0). Afterwards, SNP clusters and SNPs close to indels were removed using SnpSift (version 4.3 t) filter (Cingolani, Patel, et al., 2012). The summary of the variant analyses was generated by SnpEff (version 4.3 t) (Cingolani, Platts, et al., 2012).

### 2.4 Visual inspection

The vcfR package (version 1.12.0) was used to read and visualize the vcf file for quality control (Knaus & Grünwald, 2017). The read depth (DP), the mapping quality (MQ), the Phred‐scaled quality (QUAL), and the variants were visualized. This identified several low confidence areas with very high DP. The masker() function was used to filter out data outside the confidence range: DP (0–2500) and MQ (50–60).

### 2.5 Phylogeny of *A*. *solani* samples and extraction of SDHI gene data

In order to validate the nature of our samples, we extracted three commonly used barcode genes, *gapdh*, *rbp2*, and *tef1*, that can be used to distinguish *Alternaria* spp. (Woudenberg et al., 2013). References for *gapdh* (KC584139), *rbp2* (KC584430), and *tef1* (KC584688) were extracted form NCBI search against the reference genome using BLAST+ (Camacho et al., 2009). The chromosome number, start, and stop coordinates from the BLAST output were extracted and stored as an interval file. Then the GATK tool called FastaAlternateReferenceMaker was used to replace the reference bases with the alternative bases at variant sites available in the vcf files over the specified interval (McKenna et al., 2010). The intervals were as follows (chr: start stop): GAPDH; CP022029.1:862512–863090, RPB2; CP022028.1:224005–224869 TEF1; CP022026.1: 1668499–1668833. All sequences were loaded in ab12phylo (Kaindl et al., 2021), and a multi‐gene phylogeny was constructed using built‐in RaxML‐NG (Kozlov et al., 2019) including *A*. *solani* reference sequences and the sequences of two closely related sister species *A*. *dauci* and *A*. *porri* (GAPDH (KC584111, KC584132), RBP2 (KC584392, KC584421), TEF1 (KC584651, KC584679) for *A*. *dauci* and *A*. *porri* respectively). To analyse the genomic regions coding for the SDH subunits, we performed a BLAST search of sequences each of the SDH subunits against the *A*. *solani* reference and used the identified start and stop site to extract alternative reference fasta files using GATK (McKenna et al., 2010).

### 2.6 Genetic diversity summary statistics

Basic genomic statistics were calculated with the R package PopGenome (version 2.7.5) (Pfeifer et al., 2014). For whole‐genome analysis, vcf files were split into smaller chunks using vcftools (version 0.1.16) (Danecek et al., 2011) and imported using the readData function (include.unknown=F, format=”VCF”, SNP.DATA=T, big.data=T). Statistics were calculated via neutrality.stats() and diversity.stats() and for further use transformed into R‐dataframes. Populations were defined via the set‐populations() function. The Site Frequency Spectrum (SFS) was generated by extraction of AC‐values from the vcf file. This was done using the commands strsplit(), do.call(), and table() in base R. Histograms were plotted using ggplot2 (version 3.3.5) (Wickham, 2009).

Sliding window analysis of SNPs per site was performed using the sliding.window.transform() option of PopGenome (w=10000, j=10000, type=2, whole.data=T). To achieve that, vcf chunks of chromosomes were imported separately and analysed discretely. Therefore, a function was defined to allow the analysis of each chromosome and to re‐aggregate generated data, subsequently. This is necessary since the native function for data‐import (readVCF) does not apply for haploid datasets. Segregating sites per window were extracted and normalized per site. ggplot2 was used to visualize our findings.

### 2.7 Population phylogeny

We extracted the SNPs as the alternative reference genomes using the GATK FastaAlternateReferenceMaker tool to create an alignment of the whole isolates’ genomes and to construct a population phylogeny (DePristo et al., 2011). To create a maximum likelihood phylogeny RAxML version 8 (Stamatakis, 2014) was used with ‐p 1122590 ‐f a ‐x 1122590 ‐m GTRGAMMA ‐# 100 ‐s input ‐n output and 100 bootstraps. The phylogenetic tree was visualized using the R packages ggtree version 1.16.6 (Yu, 2020) and treeio version 1.8.2 (Wang et al., 2020).

### 2.8 Principal Component Analysis (PCA)

As a dimensionality‐reduction technique, a principal component analysis (PCA) was performed using the R packages gdsfmt (version 2.18.0) and SNPRelate (version 1.18.1) (Zheng et al., 2012). GGplot2 (version 3.3.5) was used for visualization. To avoid the overlapping labels on the PCA, the R package ggrepel (version 0.9.1) was used (Slowikowski, 2018).

### 2.9 LDHelmet

To test for signals of recombination on the genome, measured as the recombination rate in ρ per bp, we used LDHelmet (Chan et al., 2012). Like described previously (Stam et al., 2019), fasta sequences were retrieved for each isolate using FastaAlternateReferenceMaker from GATK (McKenna et al., 2010). Next, LDHelmet was executed looping over each of the chromosomes in windows of 50 SNPs, using recommended parameters for the recombination search space. We specified a burn in of 100,000 iterations and 1,000,000 true iterations and a block penalty of 50. The output was visualized using ggplot2.

### 2.10 LEA

Ancestry analysis was performed using the R package LEA (version 3.2.0) (Frichot & François, 2015). For that, vcf files were converted into a genotypic matrix using vcftools (‐‐plink) (Danecek et al., 2011) and the LEA‐function “ped2geno”. This file was then transformed manually (9=1, 2=0). In the LEA package, *sparse nonnegative matrix factorization* (sNMF) was performed and a cross‐entropy criterion was calculated to identify the best statistical model describing ancestral populations of the dataset. The minimal cross‐entropy for the dataset was determined with multiple repetitions (K1:15, ploidy=1, entropy=T, rep=10). Admixture analysis was performed for k=6, k=7, and k=8 [*Q(obj*, *k*, *run*=*which*.*min(cross*.*enropy(obj*, *k)))*] and ordered according to individually assigned genotypes or sample origin. Visualization and inference with mutations was done using the tidyverse package (version 1.3.1) (Wickham et al., 2019) and ggplot2.

### 2.11 poppR

A Minimum Spanning Network (MSN) analysis was performed using poppR (version 2) (Kamvar et al., 2014, 2015). Whole‐genome data was extracted from the vcf file using vcfR (version 1.12.0) (Knaus & Grünwald, 2017), omitting sites with >2 alleles called. Ploidy was set to 1, and distances between the samples were calculated using dist(). The MSN was calculated using the function poppr.msn() and plotted with plot_poppr_msn().

### 2.12 Splitstree

Phylogenetic trees were generated with splitstree (version 4.17.1) (Huson & Bryant, 2006). Aligned fasta sequences were retrieved for each isolate using FastaAlternateReferenceMaker from GATK (McKenna et al., 2010) and used for network construction using default settings in splitstree.

### 2.13 Distance matrix

An SNP distance matrix was constructed from the alternative reference sequences with the extract alternative reference fasta option from GATK (McKenna et al., 2010). Two versions were created. One including all sites, one removing all incomplete cases (e.g., removing sites that were called in one isolate, but not in another). The matrix was created using snp‐dists ‐a (https://github.com/tseemann/snp‐dists).

### 2.14 Data availability

Raw short read data are deposited to NCBI SRA (project number PRJNA746421). SNP call files are deposited to Zenodo.org under doi 10.5281/ (Stam et al., 2021).

## 3 RESULTS

### 3.1 *A*. *solani* is highly polymorphic across Europe

To assess the genome‐wide diversity of *A*. *solani* in Europe, we selected samples to represent a cross section of the continent. In Europe, 43 isolates were collected from potato fields – 8 from Belgium, 8 from Northern Germany (Lower Saxony), 7 from Southern Germany (Bavaria), 8 from Serbia, and 12 from Sweden (Figure 1, Table 1). We also included five isolates originating from the United States. All isolates were sequenced using DNBSeq and had an average read depth of 62x coverage, after mapping and filtering (Table 1). On average we found 47,098 SNPs in a single isolate, when comparing to the reference, corresponding to one SNP every ~700 bases. The number of SNPs in most isolates ranged from 32,079 SNPs (in DE_NM019) to 52,911 (in DE_NM017). BE_SL002 appeared to be an outlier with 157,798 SNPs compared to the reference. However, extraction of the sequences of typical barcode markers used in phylogenetic analyses of *Alternaria* species (RPB2, GAPDH, and EF1) confirmed that BE_SL002 is *A*. *solani*. With this combination of barcodes, we can define two haplotypes (seen as two major branches in Figure S1A). BE_SL002 fits firmly within one of the two haplotypes and does not cluster with one of the related sister species. Alignment with the reference sequences for GAPDH as provided by Adhikari et al. (2020) reveals that all of our isolates belong to what they identified as the AlAsHs4 haplotype, which they assigned to potato hosts (Figure S1B).

In total, we found 262,928 (136,180 without BE_SL002) SNPs between all isolates. Due to the comparably large number of unique SNPs in BE_SL002, further analyses were done without this sample, to allow better comparison.

We found a transition versus transversion (ts/tv) ratio of 3.05. Forty‐eight percent of the SNPs are singletons. The Site Frequency Spectrum (SFS) for the sample set shows a homogenous decrease first. However, the overall number of SNPs is not decreasing continuously, in fact, some minor peaks are occurring for higher SNP frequencies (Figure S2). This indicates that there are no large population expansions (as would be the case with increased number of singletons). The slightly uneven distribution might be an effect of partial clonality in the sample set. Next, we calculated several population genetics summary statistics for the sample set. Overall, Watterson θ per site equals 0.019 and π equals 0.016. The overall Tajimas’ D of the dataset is rather neutral with a value of 0.629.

On average we found one SNP every ~241 bp. SNP density varies per chromosome, ranging from one every 197 bp for chromosome 4 to one every 277 bp for chromosome 2. Using sliding window analysis with 10kb windows, confirmed that SNP‐density varies along the chromosomes and is highest and lowest in chromosome 6 and 1 respectively (Figure S3). The different genes coding for the different SDH subunits that are known to be subject to fungicide resistance mutations are located on chromosomes 7 (*SdhB*), 2 (*SdhC*), and 5 (*SdhD*). Thus, the fungicide target genes are not located specifically on very conserved or very diverse chromosomes.

### 3.2 *A*. *solani* shows differences in diversity between locations

Next, we wanted to know whether the genetic variation is similar for each of the five sample locations in Europe, and in our US samples. To this end, we summarized all statistics per location (Table 2). Even excluding BE_SL002, the Belgian samples had the highest number of SNPs (103,971 SNPs in total), followed by Bavaria (101,623). Lower Saxony and Serbia have 62,378 and 63,997 SNPs respectively. Interestingly, in Sweden, from where we analysed 12 samples, rather than 7 or 8, the number of segregating sites is only 71,232. This might in part be due to the fact that the Swedish samples were all collected in one locality, but it should be noted that with the exception for 1 sample this was also the case for Serbia. Moreover, a clear link between the number of segregating sites and the geographical spread between the samples of a certain locality cannot be made. To illustrate, the furthest distance between samples in Belgium is 107 km, the maximum distance between samples in Lower Saxony is 262 km, yet Belgian isolates remain more diverse.

**TABLE 2:** Diversity statistics per locality

Country	Segregating Sites	Theta Watterson per Site	Theta Pi per Site	Tajimas’ D	Clones (n)	Samples
Bavaria	101623	0.0221	0.0227	0.1611	1 (2)	7
Belgium	103971	0.0226	0.0234	0.213	0 (‐)	7
Lower Saxony	62378	0.0128	0.0118	−0.4428	2 (2, 3)	8
Serbia	63997	0.01315	0.01312	−0.00956	2 (2, 4)	8
Sweden	71232	0.0126	0.0153	1.0242	3 (3, 2, 2)	12
USA	55964	0.0143	0.0135	−0.4143	1 (2)	5
Total	136180	0.0164	0.0192	0.6295	‐	47

In line with the higher number of SNPs, we also found that the Belgian samples have the highest nucleotide diversity (Watterson's θ or π). Interestingly, we found some variation in Tajima's D. However, Tajima's D is negative or close to 0 in most localities. This indicates a relatively high number of singletons and is in line with the overall observations from the SFS. However, a Tajima's D of 1.024 in Sweden seems to violate the assumption of neutral theory suggesting less low‐frequency polymorphisms, probably due to higher clonality in these samples.

### 3.3 Signatures for sexual recombination

*A*. *solani* is considered to be an asexual pathogen, but some reports suggest parasexual reproduction is occurring in different populations and maybe even between related species (Alvarenga et al., 2016; Meng et al., 2015; Zhao et al., 2021). Therefore, we looked for signatures for recombination using LDHelmet (Chan et al., 2012). These analyses show clear evidence for recombination on the genome, indicated by high ρ/bp peaks, suggesting that parasexual recombination is likely, though signals on some of the chromosomes are very low (Figure S5). Next, we looked specifically at the chromosomes that harbour the genes coding for the two *Sdh* subunits with the most mutants detected. The gene for *SdhC* lies on chromosome 2, at position 3,213,044. Chromosome 2 contains only two major peaks at 2,883,609 and 3,851,760. Such limited number of possible sites of recombination and them being 500,000 bp away from the gene indicate that there is no specific recombination around these sites. The gene coding for *SdhB* lies on chromosome 7 at position 1,345,474. Chromosome 7 only has lower ρ/bp peaks, and we see no indication that fungicide resistance mutations affected the recombination around the *SdhB* locus as compared to other loci (Figure S5).

### 3.4 Classification of *A. solani* genotypes

**FIGURE 2:**
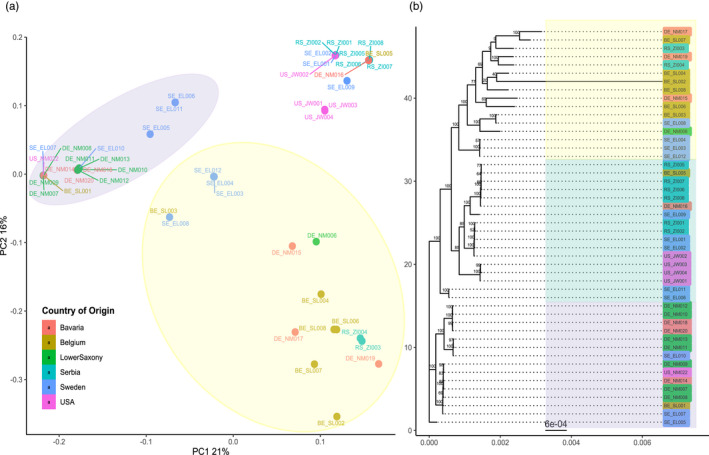
Principal component analysis (PCA) and phylogenetic tree constructed of the 48 *Alternaria solani* isolates. (a) Scatter plots of the first two principal components made using SNPRelate and ggplot2 packages in R. The x‐ and y‐axis represents the PC1 (with variance explained 21%) and PC2 (with variance explain 16%). The isolates are colour‐coded according to their region of origin. Highlighted clusters in yellow, green, and purple are based on the clustering on the phylogenetic tree. (b) Phylogeny of reconstructed whole‐genome sequences for each of the 48 isolates, made using RAxML (GTRGAMMA) with 100 bootstraps. The phylogenetic tree is constructed using treeio and ggtree packages in R. The colour coding of the isolates corresponds to the region of origin. The x‐axis and scale bar indicate the branch length

We hypothesized that there is variation in genetic diversity and the genetic background of the isolates between each locality. Therefore, we wanted to define the overall population structure and see whether genotype distribution of *A*. *solani* matches with respective sample origin, for example, genotypes showing regional clustering. We first reduced the complexity of the data for PCA analyses. Combined the first three components explain 49% of the variation (Figure S4). The plot of the two first principal components (Figure 2A) shows three or four clusters, each of varying size and spread. All clusters that can be identified contain isolates from different geographical origins. When plotting PCA 1 versus 3, it can be seen that BE_SL002 is the only sample driving the difference on the third principal component and thus a clear outlier (Figure S4 (B)). To further corroborate these findings, we set to construct a phylogenetic tree based on all identified SNPs in the isolates (Figure 2B). As expected, isolates that grouped close together in the PCA also group in the phylogenetic tree. These findings are supported by high bootstrap values. In the phylogenetic tree, BE_SL002 groups together with the isolates that are closest to it in the PCA, yet it has a long branch length. Colour coding by locality in the PCA and on the branches of the tree shows that most localities indeed harbour several unrelated isolates.

**FIGURE 3:**
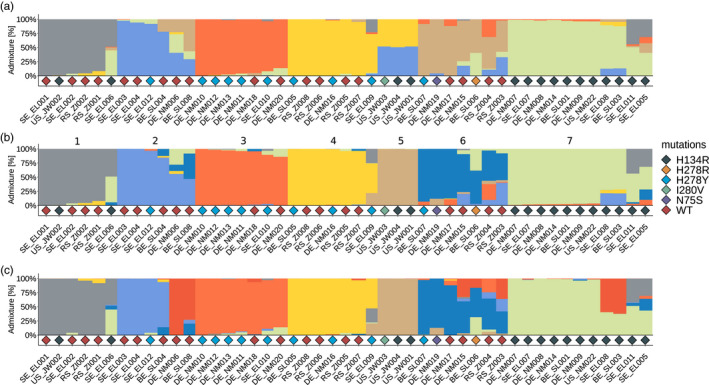
Ancestry analysis on *A*. *solani* samples. Ancestry analysis was performed on *A*. *solani* isolates (n=47, BE_SL002 excluded) using the R‐package LEA [*snmf(K*=*1*:*15*, *rep*=*10*, *ploidy*=*1)*] for 6 (a), 7 (b), and 8 (c) ancestral populations. Colours represent individual genotypes assigned by *sparse nonnegative matrix factorization*. Bar plots show the admixture of each genotype in every isolate. Diamonds represent mutations against SDHIs found in the data set. The numbers in panel B denominate the assigned genotype (GT) as used in Table 1 and other parts of this manuscript

To further define the population structure of the isolates, we calculated admixture using the R package LEA (excluding BE_SL002). To estimate the most likely number of ancestral populations of the dataset, the cross‐entropy criterion was calculated. The lowest cross‐entropy was observed at K=7 (Figure S6), thus admixture analysis was conducted for K = 7 populations as well at K=6 and K=8. With K = 7, we can see groupings similar to those observed in the PCA. Several clusters show mainly uniform, un‐admixed ancestry. For example, the samples BE_SL005, RS_ZI008, RSZI006, and DE_NM016 (all yellow in Figure 3B) cluster closely together in the PCA and form a monophyletic clade in the phylogeny. All genotypes are present in distinguishable groups. When comparing K = 6 and K = 7, we observe one cluster (US_JW001, US_JW003, US_WJ004) dividing into clear not‐admixed genotypes. In some samples, increasing K can be associated with increasing admixture of various genotypes (e.g., in DE_NM006). For K=7, 13 isolates show a dominant ancestor only with minimal admixture. Eighteen isolates show admixture of 2 ancestors and 16 samples show admixture with 3–4 ancestors. Admixture from five ancestors can only be found in RS_ZI004. Note that the results of this whole‐genome genotyping do not correlate with the haplotyping results for the three barcode genes, suggesting the three barcodes alone cannot accurately capture the diversity of *A*. *solani* in a field (Figure S1, Table 1).

Next, we assigned each isolate to the respectively most dominant of the seven genotype groups and calculated diversity statistics for each of them (Table 3). The nucleotide diversity differs between each genotype group. Genotype 6 (dark blue) has the highest numbers of SNPs, 96,239, probably because it is also the most admixed. Interestingly, the number of SNPs of the genotypes does not scale with sample size, for example, genotype 3 (n=7) has only 14,459 SNPs. Therefore, the nucleotide diversity per site diverges strongly between the genotypes, ranging from 0.002 (genotype 4) to 0.023 in genotype 6. Tajimas’ D is ranging from −1.709 in genotype 4 up to 2.212 in genotype 3. Genotypes 2, 6, and 7 show neutral Tajimas’ D; the calculation for Genotype 5 is not possible due to sample size.

**TABLE 3:** Diversity statistics per genotype

Spalte1	Genotype	Segregating Sites	Theta Watterson per Site	Theta Pi per Site	Tajimas’ D	Clones (n)	Samples
1	Genotype 1	21461	0.00549	0.00459	−1.24863	1 (5)	6
2	Genotype 2	72352	0.0169	0.0169	0.0182	1 (3)	6
3	Genotype 3	14459	0.00314	0.00432	2.21246	2 (2, 5)	7
4	Genotype 4	15640	0.0034	0.00242	−1.709	1 (6)	7
5	Genotype 5	933	0.000331	0.000331	NA	1 (2)	3
6	Genotype 6	96239	0.0224	0.0229	0.142	0 (‐)	7
7	Genotype 7	44435	0.00808	0.007	−0.65001	1 (7)	11

Seeing the limited diversity in some genotype groups, we asked how many isolates could be considered clones of each other. To this extent we created a pairwise SNP‐distance table for isolates (Table S1). This reveals that on average there are 40,282 SNPs between the isolates (35,365 when taking only complete cases (e.g., sites that have been called in all isolates reliably). The lowest number of SNPs between two isolates is 822 (135 with complete cases). Pairwise comparisons with such low number of differences suggest the existence of true clonal isolates. Looking in the genotype groups constructed by LEA, we see that 4 genotype groups contain 3–4 samples that have less than 1500 SNPs between them, and one genotype group shows 2122 SNPS shared between 6 of its samples. Thus, our Europe‐wide sample set consists of a considerable number of likely true clones.

To better visualize the diversity per location, we also sorted the isolates per locality (Figure S7). It should be noted that each location contains a mix of different genotypes. The dominating genotypes of a locality vary slightly, but it is not possible to assign a single dominant genotype to a single region, thus, refuting our hypothesis that *A*. *solani* genotypes would show regional clustering. Variation in the diversity within geographical regions can also be observed. The samples from Belgium and Sweden show the most complex admixture structure, followed by Bavaria. The high complexity in Sweden is particularly interesting, because in absolute number of segregating sites, this population did not appear to be the most diverse. Interestingly, some of the clones described above can be found on different sides of the European continent (e.g., BE_SL005 clusters with some of the Serbian isolates) or even across continents (US_JW002 clusters with Serbian and Swedish isolates).

### 3.5 SDHI mutations arose independently in different genotypes

To understand the rise and spread of SHDI fungicide resistance, we overlaid the genotyping data with our data on SDHI‐target mutations. The H134R and H278Y mutations occur 15 and 10 times in our dataset, respectively. H134R is found in every locality, except Serbia; H278Y can be found in all locations but Serbia and the United States. Furthermore, we found three mutations (H278R, I280V & N75S) that occur only once in the dataset. In 20 samples, we found no mutation related to SDHI fungicides. Mutation‐free isolates were collected in all different localities except for the United States.

Overlaying the data shows that the mutations are not linked to the background genotypes that we defined previously. The different *sdh* SNPs were found in different genetic backgrounds (Figure 3B). For example, H134R occurs in the grey, brown, and green genotype. H278Y occurs in the blue, red, and yellow genotype. This strongly indicates that mutations in the SDH subunits arose independently in different genetic *A*. *solani* backgrounds.

**FIGURE 4:**
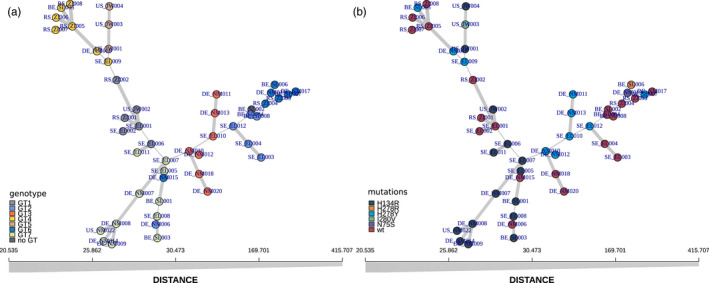
Minimum spanning network analyses (MSN) for our *A*. *solani* samples. MSN showing the relatedness of the samples as produced by the R package poppR. Both panels show the same network, with identical scaling (x axis). Each circle represents an individual isolate. Colours in (a) correspond to previously assigned genotypes, colours in (b) highlight the isolate's SDHI mutations

To obtain additional lines of evidence for our findings, we constructed a minimum spanning network (MSN) based on all SNP data (Figure 4). These networks confirm that the LEA method reliably assigned genotypes to our isolates. Only two small differences can be observed in the MSN (Figure 4A). Isolates DE_NM006 and DE_NM015 appear to sit within the samples assigned to genotype 7 rather than genotype 2 and genotype 6 respectively. Looking at the results from LEA, it becomes evident that these two samples are in fact the two most admixed samples, and therefore the placement of these samples on the MSN should be taken with some caution. When looking at the occurrence of the SDHI mutations on the MSN (Figure 4B), the individual mutations can be seen on the different branches.

**FIGURE 5:**
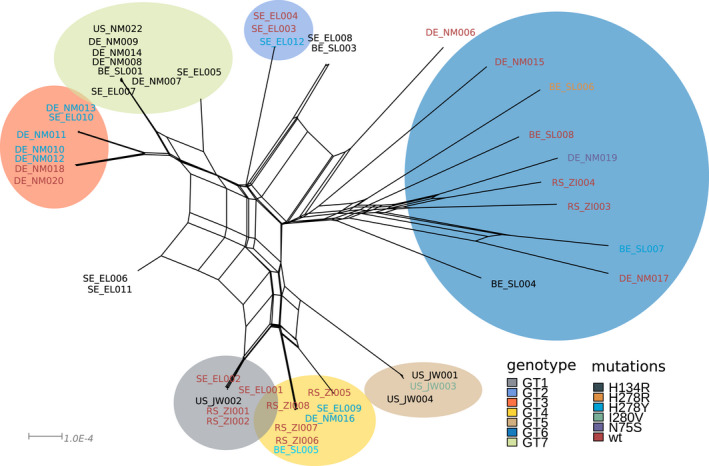
Splitstree analyses for the A solani isolates. Splitstree analysis reveals the same genetic clusters as our ancestry and MSN analysis. Several isolates show clear admixture or signals of recombination, as shown by the lines connecting the individual branches. The circles represent the dominant genotype as assigned with LEA, isolates that have no coloured background are admixed. The text colour for our each isolate indicates the respective SDHI mutation of the isolate

As a final line of evidence, we used splitstree (Huson & Bryant, 2006) to analyse the population structure of *A*. *solani* (Figure 5). Splitstree also shows a clear separation of the seven previously assigned genotype groups, with the most admixed isolates positioned between the groups. Moreover, it clearly shows evidence for recombination between some of the isolates, as illustrated by the connections between some of the branches. The phi test for recombination confirms that there is significant evidence for recombination. Lastly, the analyses also suggest that, as we observed with LDHelmet, the different *Sdh* mutations did not spread through recombination. Similar to the MSN analyses, Figure 5 clearly shows that the H134R and H278Y mutations appear multiple times at different branches. These branches are not connected through recombination events, and thus we conclude that they arose independently.

## 4 DISCUSSION

In this study we present the first Europe‐wide genetic diversity study of the early blight pathogen *Alternaria solani* based on whole‐genome data. We used 43 isolates collected in five different localities ranging from Sweden to Serbia. Seeing that *A*. *solani* is considered a clonal pathogen (synonymous: mitosporic fungus, Deuteromycete, *fungi imperfecti*), we expected to see certain geographical patterns related to the genetic diversity, for example, patterns of isolation by distance or predominance of a certain genotype in a locality. The strongest geographical patterns can be observed in invasive pathogens, like the rice and wheat blast fungus *Magnaporthe oryzae*, where multiple genetic lineages can be found in Southeast Asia, but single invasive lineages spread elsewhere and sometimes became epidemic (Gladieux et al., 2018; Islam et al., 2016). However, clear geographical patterns in population structure can also be observed in cosmopolitan pathogen species like the oomycete potato pathogen *Phytophthora infestans*, the causal agent of late blight, or in other ascomycete pathogens such as the sugar beet pathogen *Cercospora beticola* (Knight et al., 2019) and the well‐studied wheat pathogen *Zymoseptoria tritici* (Vagndorf et al., 2018).

We did not detect any geographical clustering of *A*. *solani* isolates in this study. This could be an artefact of the relatively small sample size per locality. However, some pathogens do already show clear separation even when taken such small samples (Mariette et al., 2016). High genotype diversity and lack of isolation by distance has previously been observed for *A*. *solani* in an SSR (simple‐sequence repeats)‐based study with populations from China. Assumed clones were detected in multiple populations separated by thousands of kilometres and random association among loci was found in half of the populations assayed (Meng et al., 2015). Our whole‐genome data now suggest that these results were not an artefact of the low‐resolution markers used.

Such lack of isolation by distance has been seen as well for several other supposedly clonal ascomycete pathogens. On a regional scale similar to our sample sites in Germany, this has been observed for the Brassica pathogen *Leptosphaeria maculans* in a collection from northern and southern France (Travadon et al., 2011) and on a global scale for the barley pathogen *Ramularia collo*‐*cygni* (Stam et al., 2019). *Vice versa*, isolates from distant sites cluster together on the phylogenetic tree, suggesting long distance transport, for example, by wind or seed tubers.

Adhakiri et al. compared field population diversity of three *Alternaria* species on potato and tomato in North Carolina and Wisconsin and found that *A*. *solani* had relatively lower diversity than *A*. *alternata* and *A*. *linariae*. Yet, four different haplotypes were found in one field and haplotype divergence was found in all three species (Adhikari et al., 2020). Our analyses revealed up to nine definable genotypes in *A*. *solani* in our sample set. Deeper analyses showed that some of the collected isolates from these genotypes are at least likely purely clonal. They contain very few SNPs between them. Whether such clones perform worse or better under specific climatic conditions or on specific cultivars remains to be investigated.

Based on the results of our study, we conclude that the mutations in the genes coding for the different SDH subunits that lead to resistance or higher tolerance to SDHIs can be found in multiple genetic backgrounds. This indicates that the mutations occurred multiple times at different locations, rather than that a single resistant isolate emerged and spread throughout Europe. Evolution of tolerance against QoI fungicides was initially suggested to have arisen once, due to its association with an arbitrarily assigned genotype (GT I), and then spread. However, recent work showed that the F129L mutation in the cytochrome b target gene responsible for this resistance could since 2016 also be observed in another genotype (Nottensteiner et al., 2019). Our study further confirms the genetic heterogeneity of QoI tolerant isolates, as all of the isolates in this study show the F129L mutation. As a consequence, all isolates with SDHI mutations possess dual fungicide resistance, which makes controlling the pathogen increasingly difficult.

A recent study in *Z*. *tritici* concluded that azole resistance is likely the result of so‐called “hotspot evolution” with convergent changes in small sets of loci and more population‐specific allele frequency changes (Hartmann et al., 2020). Fungicides can form a major bottleneck for fungal populations, but the ability to recombine allowed population‐specific adaptation in *Z*. *tritici* populations. Even though we found that fungicide resistance or fungicide tolerance mutations arose multiple times independently, future studies looking deeper into the effects of fungicide induced bottlenecks in *A*. *solani* are now required.

One additional outcome of our study is clear evidence for recombination. Previous studies have suggested (para‐)sexual recombination needs to happen in the field in order to explain the observed diversity patterns (Alvarenga et al., 2016; Meng et al., 2015; Zhao et al., 2021), but the use of markers with low resolution left this open for confirmation. We used whole‐genome data and three different methods to test possible recombination. All methods indicate that the observed genetic diversity arose in part through recombination. Recombination events likely make is easier for fungicide resistances to be spreading faster through different genetic backgrounds. This, combined with the fact that true clones can be found across continents, potentially amplifies the spread of more aggressive genotypes. For example, Bauske et al. (2017) already found that *A*. *solani* isolates possessing the *SdhD*‐D123E mutation were significantly more aggressive *in vivo* compared with wild‐type isolates.

Fungicide pressure and the aggressiveness of *A*. *solani* isolates under certain conditions or on a certain host cultivar alone might not be the only determinants for *A*. *solani* diversity in the field. Early blight lesions often appear to contain a mix of *Alternaria* species. *A*. *solani* often co‐occurs with *A*. *alternata* (e.g., Zheng & Wu, 2013). Tymon et al. also isolated several other small and large spored species from lesions on potato fields (Tymon et al., 2016). And also in tomato early blight symptoms can be associated with multiple species, the large spored *A*. *solani*, *A*. *linariae*, and *A*. *grandis*, and the small spored *A*. *alternata* (Bessadat et al., 2017). Ding et al. found, that in some years the disease severity in the field is more strongly correlated to the presence of early inoculum *of A*. *alternata* (Ding et al., 2020). Using barcode‐sequencing, they found that *A*. *alternata* possessed a greater diversity in the field (Ding et al., 2020). In all studies mentioned above, the disease phenotypes of *A*. *solani* isolates were more severe and *A*. *solani* is the more dominant partner in the interaction. Studies on Alternaria leaf spot on rape seed showed that also this disease is not caused just by *A*. *brassicae*, but that it can also be caused by *A*. *japonica* and that the ratio between the species and aggressiveness of the species was temperature dependent (Al‐lami et al., 2020). Findings like these indicate that genotypic diversity of *A*. *solani* can potentially also be shaped by the presence of diverse *Alternaria* species and that further research is needed to understand the effects of coinfection on early blight epidemiology. The presence of different co‐infecting *Alternaria* spp. might cause different evolutionary pressure on the isolates.

We presented a first genome‐wide diversity analysis for the early blight pathogen *A*. *solani* in Europe. We revealed surprising genetic diversity patterns throughout Europe and show that fungicide resistance evolved multiple times independently, rather than evolving once followed by spreading. These findings can help inform policy and fungicide management practices. The finding of multiple fungicide resistance mutation events highlights that there is “not one person to blame” for the rise of fungicide resistance in *A*. *solani*. This emphasizes the need to reduce the evolutionary pressure in each individual field and keeping the population size as small as possible at all times (McDonald & Linde, 2002). New strategies using evolutionary knowledge as well as the intensive application of already non‐fungicide available measures are required for good integrative disease management on a continental scale (Green et al., 2020). Only highly integrated approaches of pest management, including measures of all kinds, are capable of reducing the evolutionary potential of *A*. *solani* epidemics. To do this without increasing too much the evolutionary pressure in the field, fungicide application time frames and quantities need to be carefully adjusted as they play an important role in the development of resistances and diseases. Unfortunately, in our dataset, loss of fungicide sensitivity cannot be linked to the degree of fungicide‐use, since data is not fully available for all localities in this project.

Our analysis also shows that some true clonal isolates can be found thousands of kilometres apart. Understanding *A*. *solani* dispersal patterns over long distances will become increasingly more relevant. Recent studies have shown clear differences in aggressiveness in different *A*. *solani* isolates in general, for example, not associated with specific fungicide target mutations (Mphahlele et al., 2020). With the high‐resolution (whole genome) genotype data presented in this study, more meaningful comparisons can be made to study the link between pathogen genotype and aggressiveness. The data will also allow for studies tracing exact clones of *A*. *solani* over time, which will lead to a better understanding of pathogen dispersal patterns and should contribute to improving management strategies.

## CONFLICT OF INTEREST

The authors have no conflict of interest.

## Supporting information

Fig S1‐S7

Table S1

## Data Availability

Raw short read data are deposited to NCBI SRA (project number PRJNA746421). SNP call files are deposited to Zenodo.org under doi 10.5281/ (Stam et al., 2021).
