# Supplementary material for: Whole‐genome sequencing elucidates the species‐wide diversity and evolution of fungicide resistance in the early blight pathogen Alternaria solani
Source: Evol Appl. 2022 Feb 22;15(10):1605–20. doi: 10.1111/eva.13350 (PMC9624079; doi:10.1111/eva.13350)
Supplement: Supplementary file 1 — Fig S1‐S7 [file EVA-15-1605-s002.pdf]

Supplementary Material For:

Whole genome sequencing elucidates the species-wide diversity and evolution of fungicide resistance in the early blight pathogen *Alternaria solani*

Running title: funcicide resistance evolution in *Alternaria solani*

Severin Einspanier*[1], Tamara Susanto*[1], Nicole Metz[1], Alexander Fastner[1], Jaap Wolters[2], Vivianne Vleeshouwers[2], Asa Lankinen[3], Erland Liljeroth[3], Sofie Landschoot[4], Zarko Ivanivic[5], Ralph Hückelhoven[1], Hans Hausladen[1], Remco Stam#[1]

1 Technical University of Munich, Freising, Germany, 2 Wageningen University, Wageningen, The Netherlands, 3 Swedish Agricultural University, Alnarp, Sweden, 4 University of Ghent, Ghent, Belgium, 5 Institute for Plant Protection and Environment, Belgrade, Serbia

#Corresponding author: remco.stam@tum.de

Abstract

Early blight of potato is caused by the fungal pathogen *Alternaria solani* and is an increasing problem worldwide. The primary strategy to control the disease is applying fungicides such as succinate dehydrogenase inhibitors (SDHI). SDHI-resistant strains, showing reduced sensitivity to treatments, appeared in Germany in 2013, shortly after the introduction of SDHIs. Two primary mutations in the SDH complex (*SdhB*-H278Y and *SdhC*-H134R) have been frequently found throughout Europe. How these resistances arose and spread, and whether they are linked to other genomic features, remains unknown.

For this project, we performed whole-genome sequencing for 48 *A. solani* isolates from potato fields across Europe to better characterize the pathogen's genetic diversity in general and understand the development and spread of the genetic mutations that lead to SDHI resistance. The isolates can be grouped into 7 genotypes. These genotypes do not show a geographical pattern but appear spread throughout Europe.

We found clear evidence for recombination on the genome, and the observed admixtures might indicate a higher adaptive potential of the fungus than previously thought. Yet, we cannot link the observed recombination events to different *Sdh* mutations. The same *Sdh* mutations appear in different, non-admixed genetic backgrounds; therefore, we conclude they arose independently.

Our research gives insights into the genetic diversity of *A. solani* on a genome level. The mixed occurrence of different genotypes, apparent admixture in the populations, and evidence for recombination indicate higher genomic complexity than anticipated. The conclusion that SDHI tolerance arose multiple times independently has important implications for future fungicide resistance management strategies. These should not solely focus on preventing the spread of isolates between locations but also on limiting population size and the selective pressure posed by fungicides in a given field to avoid the rise of new mutations in other genetic backgrounds.

Keywords: Fungicide resistance, Evolution, SDHI, Early Blight, Potato, Alternaria solani

Acknowledgments: We thank Lena Forster and Regina Dittebrand for help with isolate propagation.

Figure S1

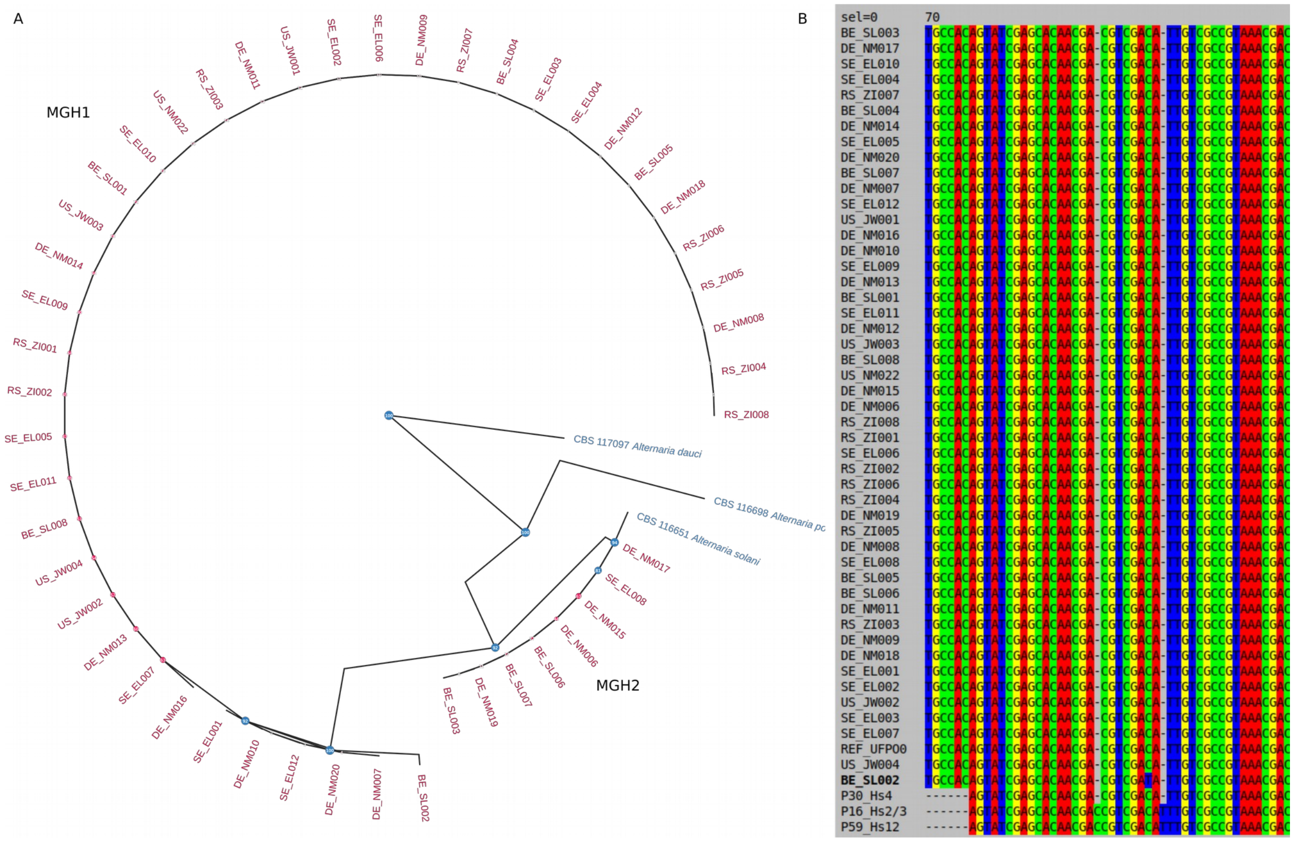

Figure S1 A) Multigene phylogeny and alignment for sequenced *A. solani* samples and selected references A) The phylogeny is based on concatenated barcode genes: *gapdh*, *rbp2* and *tef1* and selected references. All sequences were loaded in ab12phylo (Kaindl *et al.*, 2021), aligned using clustal and a multi-gene phylogeny was constructed using built-in RaxML-NG (Kozlov *et al.*, 2019) with 1000 bootstraps. Bootstrap values are indicated on all of the branches. Blue values are >70. The blue samples indicate data from reference species obtained from NCBI. MGH1 and MGH2 indicate the newly assigned haplotype based on the multiple barcode alignment used. **B)** Alignment of the defining region of the GAPDH gene as used by Adkiari et al 2020, to define *A. solani* haplotypes. The three lowest samples represent type specimens from their study and highlight identify of our isolates with P30, which was defined as haplotype AsH4.

Figure S2

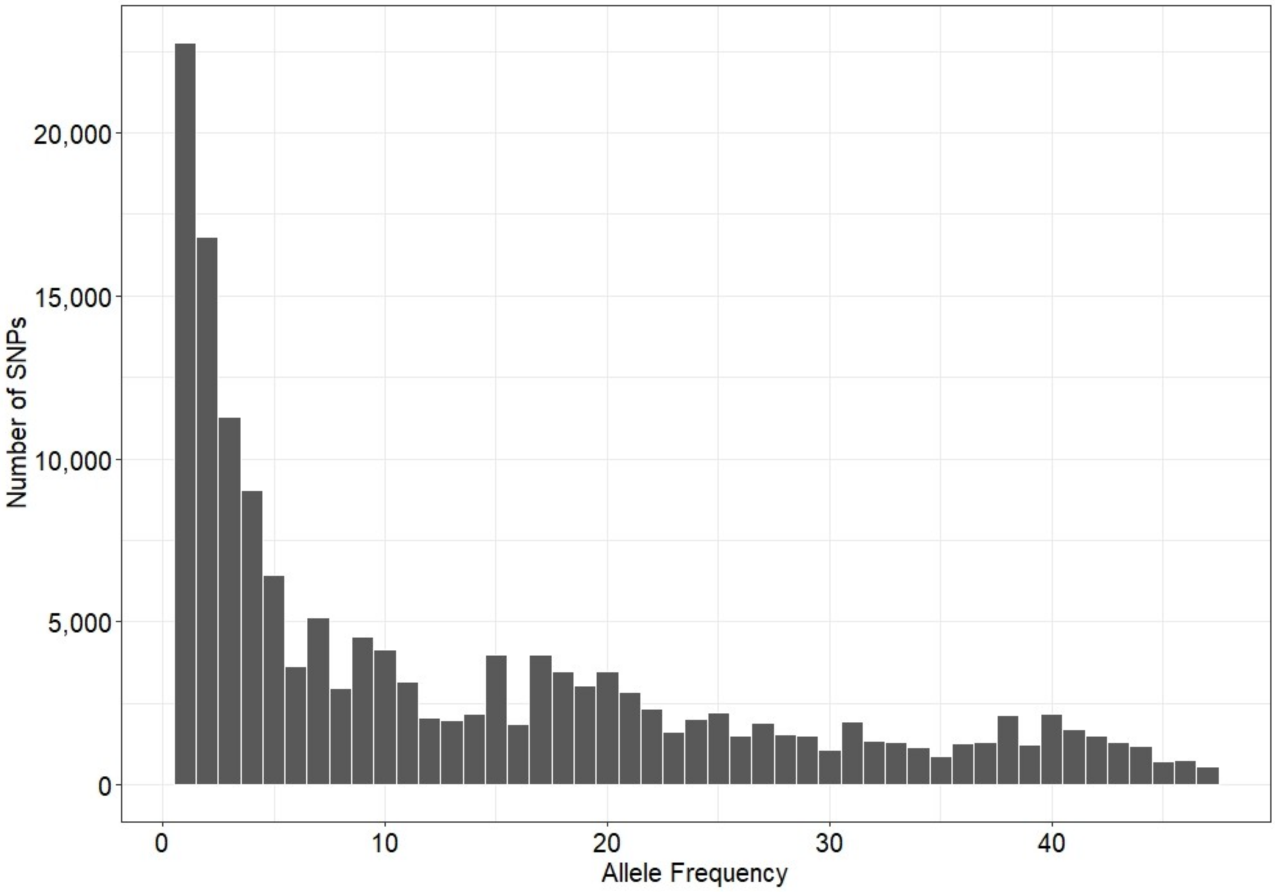

Figure S2 Site Frequecy Spectrum of all SNPs in the samples Histogram showing the allele frequencies of all SNPs in the data set (exlcuding BE:SL002). Number of SNPs is plotted (y-axis) against the frequency of the SNP in the data set compared to the reference genome.

Figure S3

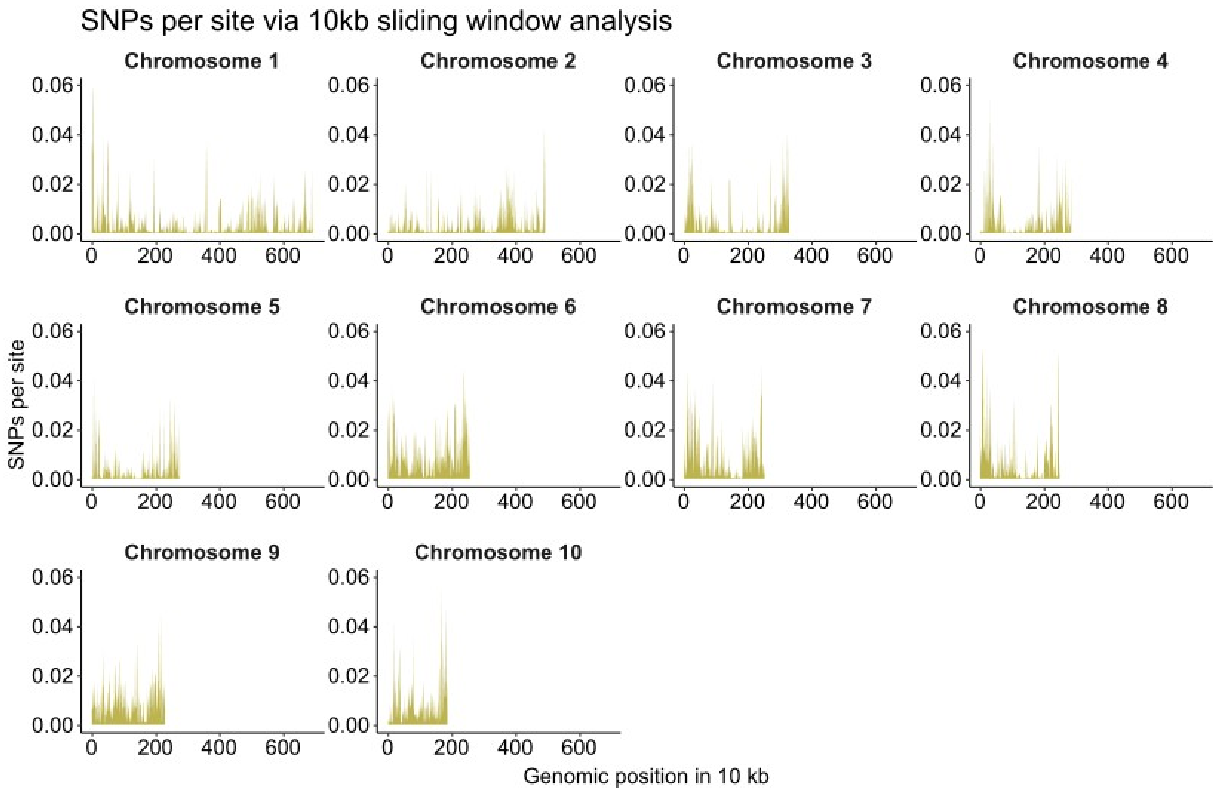

Figure S3 SNP density per chromosome SNP density for all samples, plotted in sliding windows on 10kb. Each facat represents a chromosome with the length in base pairs on the x-axis. The frequeny of Snps is indicated as SNP per site (y-axis).

Figure S4

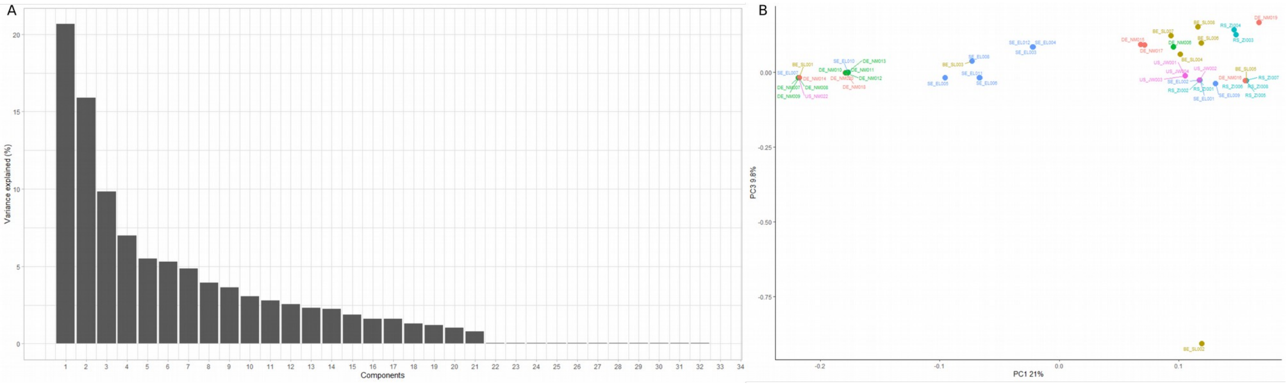

Figure S4 A) The percentage of variance explained for the proncipal component analyses. PC 1, 2 and 3 combined explain about 49% of the variation. **B) Visualisation of the data plotted on PC1 vs PC3** The sample BE_SL002 appears as a clear outlier on the lower edge of the figure.

Figure S5

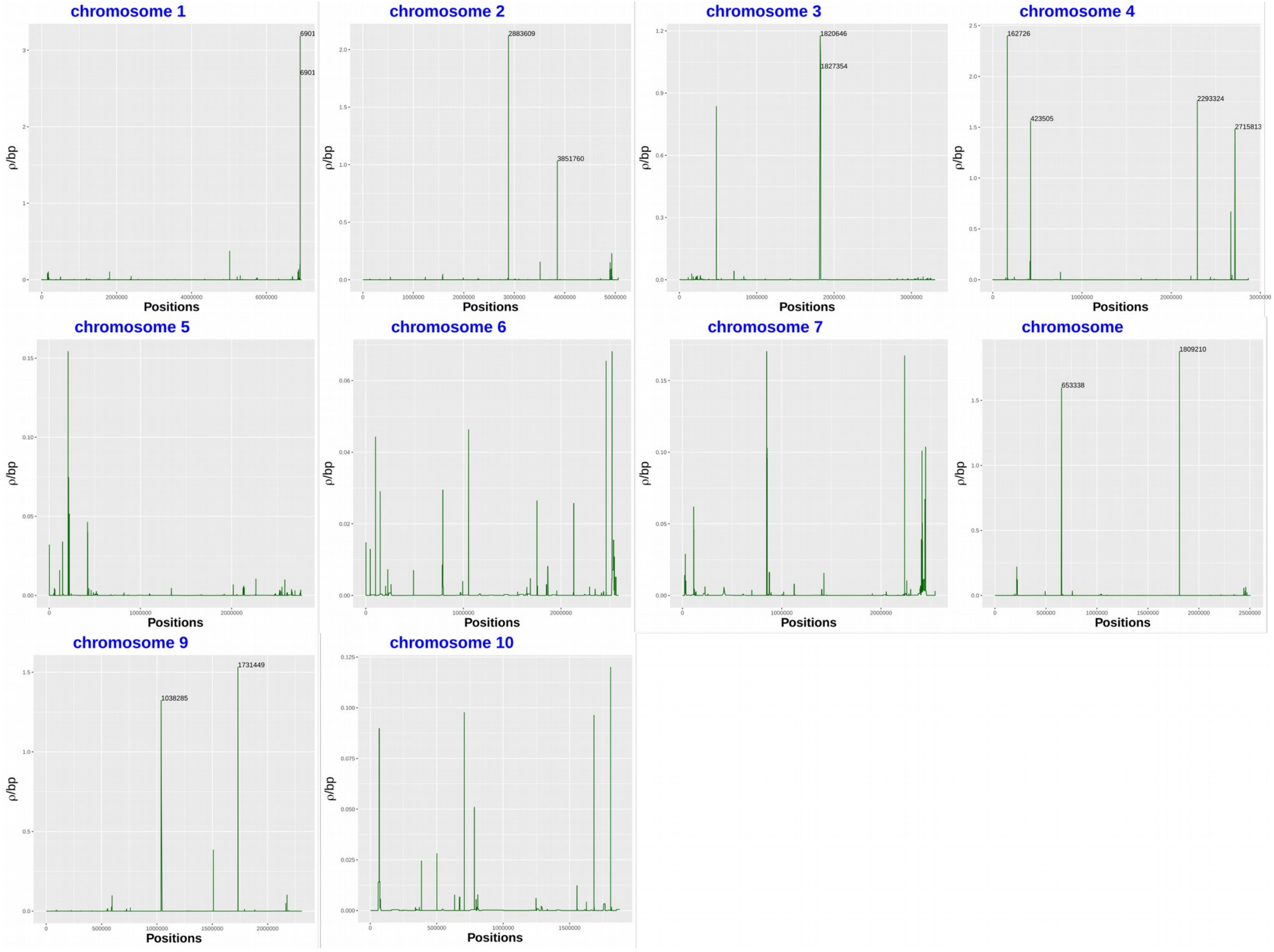

Figure S5 Recombination analyses using LDHelmet.

Recombination in r per kilobase (y axis) was calculated per position on the chromosomes of *A. solani.* The x-axis shows the scaffoldlength in base pairs. Note that the y-axis scalediffers between chromosomes and that some chromosomes show only very low signals.

Figure S6

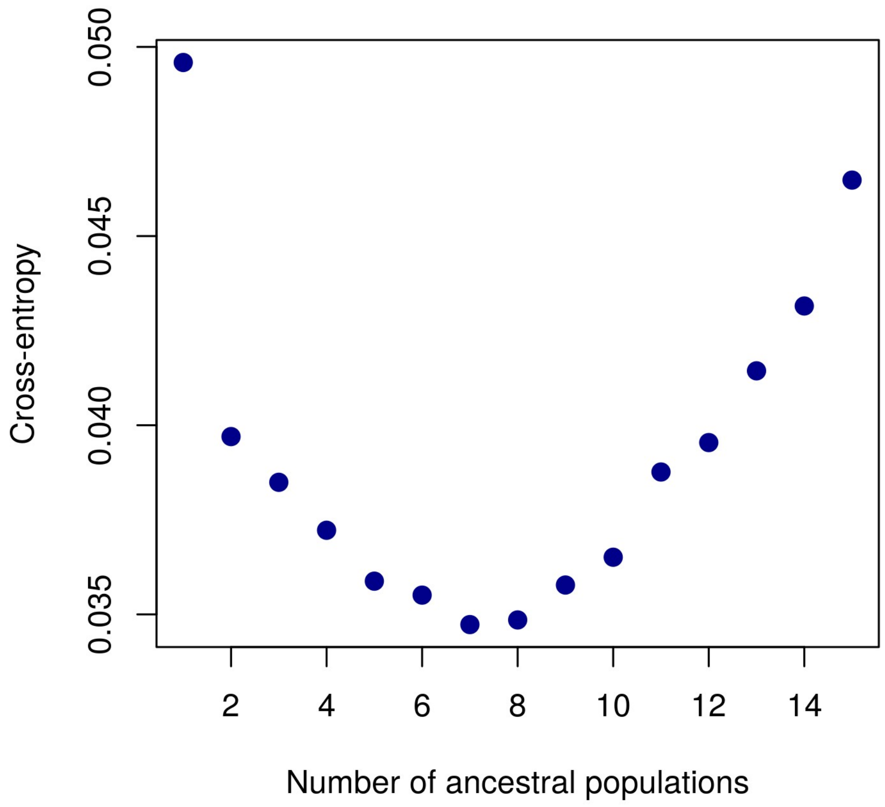

Figure S6 Cross entropy analysis in the sample set Output of the cross-entropy analyses as done with LEA. Tested were any number from 2-14 ancestral populations. The lowest value (y axis) lies at 7.

Figure S7

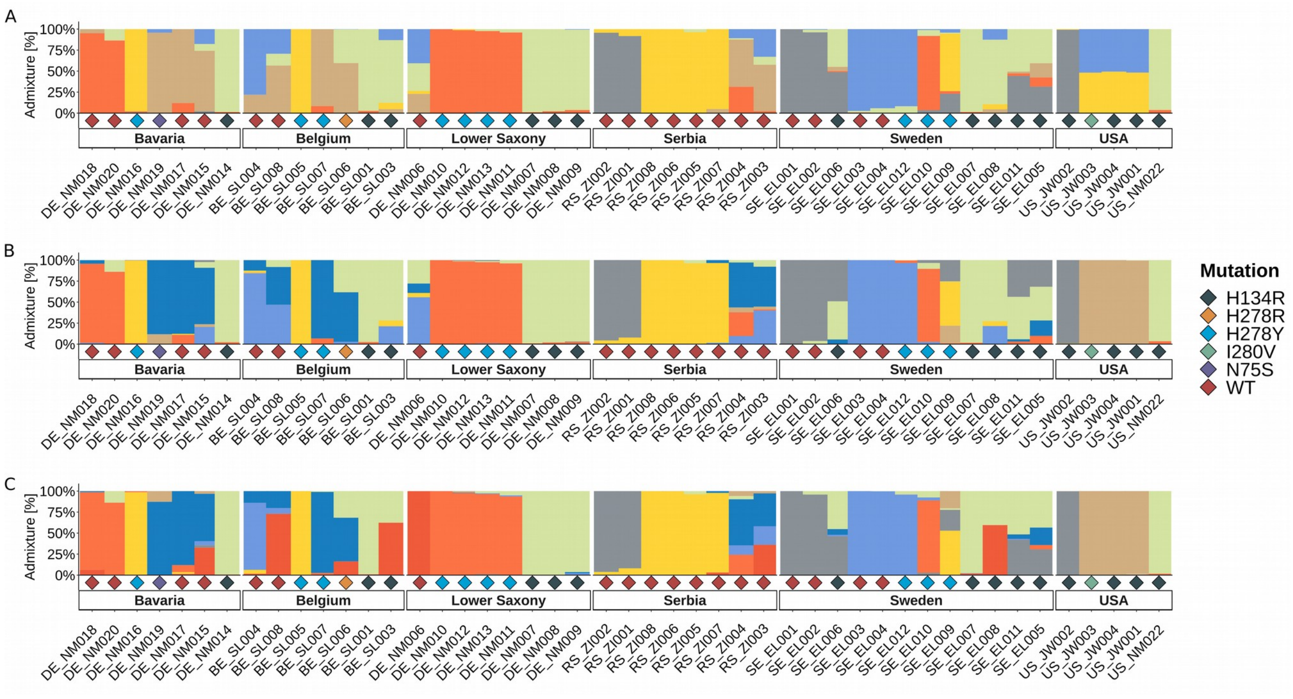

Figure S7 Genotypes and SDHI-mutations are not linked to sample locality and appear in different locations independently. Ancestry analysis was performed on *A. solani* samples (n=47) using the R-package LEA [*snmf(K=1:15, rep=10, ploidy=1)*] for 6 (A), 7 (B) and 8 (C) ancestral populations and then sorted according to sample locality. Colours represent individual genotypes assigned by *sparse nonnegative matrix factorization*. Bar plots show the admixture of each genotype in every sample. Diamonds represent mutations against SDHIs.
